# Nanobionics in Crop Production: An Emerging Approach to Modulate Plant Functionalities

**DOI:** 10.3390/plants11050692

**Published:** 2022-03-04

**Authors:** Anuj Ranjan, Vishnu D. Rajput, Arpna Kumari, Saglara S. Mandzhieva, Svetlana Sushkova, Evgenya V. Prazdnova, Sajad Majeed Zargar, Ali Raza, Tatiana Minkina, Gyuhwa Chung

**Affiliations:** 1 Academy of Biology and Biotechnology, Southern Federal University, Stachki 194/1, 344090 Rostov-on-Don, Russia; rvishnu@sfedu.ru (V.D.R.); kumari@sfedu.ru (A.K.); msaglara@mail.ru (S.S.M.); terra_rossa@mail.ru (S.S.); prazdnova@sfedu.ru (E.V.P.); tminkina@mail.ru (T.M.); 2 Proteomics Laboratory, Division of Plant Biotechnology, Sher-e-Kashmir University of Agricultural Sciences & Technology of Kashmir, Shalimar, Srinagar 190025, India; smzargar@skuastkashmir.ac.in; 3 Key Laboratory of Ministry of Education for Genetics, Breeding and Multiple Utilization of Crops, Center of Legume Crop Genetics and Systems Biology/College of Agriculture, Oil Crops Research Institute, Fujian Agriculture and Forestry University (FAFU), Fuzhou 350002, China; alirazamughal143@gmail.com; 4 Department of Biotechnology, Chonnam National University, Yeosu 59626, Korea

**Keywords:** nanoparticles, nanobionics, photosynthesis, nutrient use efficiency, sustainable agriculture

## Abstract

The “Zero Hunger” goal is one of the key Sustainable Development Goals (SDGs) of the United Nations. Therefore, improvements in crop production have always been a prime objective to meet the demands of an ever-growing population. In the last decade, studies have acknowledged the role of photosynthesis augmentation and enhancing nutrient use efficiency (NUE) in improving crop production. Recently, the applications of nanobionics in crop production have given hope with their lucrative properties to interact with the biological system. Nanobionics have significantly been effective in modulating the photosynthesis capacity of plants. It is documented that nanobionics could assist plants by acting as an artificial photosynthetic system to improve photosynthetic capacity, electron transfer in the photosystems, and pigment content, and enhance the absorption of light across the UV-visible spectrum. Smart nanocarriers, such as nanobionics, are capable of delivering the active ingredient nanocarrier upon receiving external stimuli. This can markedly improve NUE, reduce wastage, and improve cost effectiveness. Thus, this review emphasizes the application of nanobionics for improving crop yield by the two above-mentioned approaches. Major concerns and future prospects associated with the use of nanobionics are also deliberated concisely.

## 1. Introduction

Nanotechnology (NT) is witnessing exponential growth in several sectors, and it is believed to have more advancement in the near future. The global market of NT reached up to USD 42.2 billion in the year 2020 despite the global COVID-19 pandemic, and it is estimated to reach USD 70.7 billion by 2026 with a surging Compounded Average Growth Rate (CAGR) of 9.2% (Globe News Wire, 2021). The unique physicochemical features of nanoparticles (NPs) empower their significant application in crop production and food security [1,2,3]. Attaining food security by enhancing agricultural production has been a major goal for scientists and technologists across the globe.

Metal-based NPs are the most studied and found greatly useful in crop production to enhance the growth of crops and protect their health from various biotic stressors [4,5], as well as protect from abiotic stresses such as drought, temperature, humidity, and higher salt concentrations [6,7,8]. The agriculture sector has taken up the application of NPs progressively as a result of several commercial formulations such as Nano-Calcium, Magic Green (PAC International Network Co., Ltd., Köln, Germany), Nano Urea (liquid) fertilizer (Indian Farmers Fertilizer Cooperative Limited, New Delhi, India), NanoRise™ (Aqua-Yield Hub, Sandy, UT, USA), etc., are now available to enhance crop yield and qualitative traits [9,10].

The concept of nanobionics in agriculture deals with the use of NPs and their interaction with a plant’s system to improve or modulate specific functions that can notably boost crop production [11]. Nanobionics features implanting the specific NPs into plants to unleash the obscured potential that may not have been accomplished without the use of NPs. For instance, chloroplasts have a limited capacity to absorb light in the visible range, and they further limit the utilization of solar energy by almost 50% [12]. Ineptly, the photosynthetic capacity is saturated by nearly 10% of total sunlight [13]. In such circumstances, nanobionics can significantly improve photosynthesis by various means [14].

The photocatalytic ability of NPs (e.g., TiO_2_ NPs) expands the range of absorption of sunlight specifically in the near-infrared range, which is substantially helpful in improving the absorption of solar radiation by leaves, thereby enhancing photosynthesis. The process is governed by modulation of the electron transport chain, photophosphorylation, carboxylation of ribulose-1,5-bisphosphate carboxylase/oxygenase (RuBisCO), and also by preventing the aging of chloroplasts [15,16]. Enhanced expression of RuBisCO activase (rca) mRNA (by 51% compared to the control that was treated with bulk TiO_2_ NPs) has been proven to be the fundamental principle that helps in improved carbon metabolism by photosynthesis [17]. The application of nanobionics has also been effective on plants under mild heat stress by increasing the water conductance and transpiration as well as modulating the energy dissipation of photosystem-II [18].

Improved nutrient use efficiency (NUE) through controlled, efficient fertilizer uses and site-specific pesticide delivery is another unique application of nanobionics in crop production. It ensures the delivery of the active ingredient only upon receiving the external or environmental stimuli, for example pH, temperature, humidity or even manmade stimuli such as ultrasonic triggers [19,20,21]. Thus, these applications are certainly promising in terms of improving NUE as well as protecting the non-target organism and environment from inevitable exposure of agrochemicals caused by their excessive use [22].

In this review article, the applications of metal NPs as nanobionics and their application in improving crop production by modulating photosynthesis are covered. The review also deals with nanobionics as targeted and stimuli-responsive NPs that can efficiently deliver agrochemicals and control their release. The future prospect of nanobionics is deliberated by focusing on the key areas that have to be dealt in the coming time regarding socioeconomic aspects pertaining to small farmers and developing countries.

## 2. Nanobionics and Modulation of Photosynthesis

Modulation of photosynthesis is always one of the most advantageous procedures that can considerably increase the rate of crop production [23]. In addition, the notion of manipulating photosynthesis has been a source of debate for years; nonetheless, new findings have asserted that it is practically viable [24]. The efficiency of photosynthesis among C3 and C4 plants vary due to the unfavourable function of RuBisCO [25]. In terms of photosynthesis, among all the plants in the ecosystem (C3 and C4 plants), the majority (85%) of them are C3. However, the C4 plants have a better photosynthetic ability than the C3 [26]. Previously, some studies were conducted using a conventional approach to influence the function of photosynthesis by (i) improving the efficiency of the enzyme RuBisCO [27], (ii) modification of C3 plants to use the C4 photosynthesis process [26,28], (iii) manipulating the chlorophyll antenna and their size within the photosystems [29], (iv) increasing the efficiency of chlorophyll to absorb light in extended wavebands [30], and improving the photoinhibition frequency [31].

NPs as nanobionics have considerably been effective in modulating the photosynthesis capacity of plants. It helps fast-growing plants by acting as an artificial photosynthetic system, thereby improving the process [32,33]. For example, TiO_2_ NPs have been successfully studied for the potential of enhancing photosynthesis by improving the light absorption of leaves [34,35,36]. They safeguard the chloroplast from photochemical stress that might lead to ageing [37]. Recent studies have reported that TiO_2_ NPs considerably improve RuBisCO, which is one of the key enzymes helpful in improving the rate of photosynthesis [25,38]. It has also been reported to enrich the chlorophyll content in *Brassica napus* and *Solanum lycopersicum* L. [39,40].

Other metal/metal oxide NPs have also been explored as nanobionics that modulate the photosynthesis process, thereby improving the growth and overall features of the plants. In recent years, Au-, ZnO- and Ag-based NPs are being investigated for their capacity to improve photosynthesis. They mainly act on the plants by improving the chlorophyll content of the plants [41], enhancing the relative electron transport rate [42], photochemical quenching [23], and aiding the photosystem functions [43]. However, these functions are again driven by specific doses of NPs, as the higher dosage has been reported to diminish the photosynthesis apparatus [44], rate of photosynthesis [45], photosystems [46], and other associated parameters. For example, Ag NPs in the concentration ranging from 40–60 mg L^−1^ have successfully been studied for improving the chlorophyll content in the *Trigonella foenum-graecum* L. (effective dose of 40 mg L^−1^) [47], *Cucumis sativus* (50 mg L^−1^) [48], *Eschscholzia californica Cham* (25 mg L^−1^) [49], and *Helianthus annuus* L. (60 mg L^−1^). A higher dose (100 mg L^−1^) caused sharp decline (by 48.3%) in the concentration of chlorophyll [50]. Ag NPs are also reported to enhance the coefficient of photochemical quenching, thereby implying an inhibition of the electron transport chain [51].

In the case of myco-synthesized TiO_2_ NPs (50–100 nm, spherical), the dose of 25–100 mg L^−1^ could induce only a slight increase in the total chlorophyll content [52], or a dose-dependent increase [53]. However, it was reported that at 250 mg kg^−1^ of chemically synthesized TiO_2_ (15–40 nm, spherical), NPs when coupled with Plant Growth Promoting Rhizobacteria (PGPR) saw a 43.27% increase in total chlorophyll [54]. TiO_2_ NPs have excellent photocatalytic properties, and therefore 500 mg L^−1^ of TiO_2_ (2.0−5.0 nm, spherical) in combination with 210.87 mg L^−1^ of chloroplast, when coupled together to form a hybrid photosynthesis system, exhibited the highest electron transfer rate from PS-II to PS-I [55].

To further elaborate on these aspects two tables are appended below, in which Table 1 explains the application of metal NPs as nanobionics to improve the photosynthesis process, their beneficial or adverse effect, and mode of action. Table 2 includes the recent findings on metal NPs that improve or affect the plant’s chlorophyll content, their effective dose, and mode of application.

ZnO NPs are most effective NPs at the lower concentration (approximately 10–50 mg L^−1^) either by seed priming or foliar application. They enhance chlorophyll content by 40–49% and also enhance the photosystem efficiency, stomatal conductance, and CO_2_ assimilation up to 35%. ZnO nanostructures enabled nano-fertilizer enhanced growth of *Lycopersicum esculentum* L. plants via improving cellular enzyme activities [71]. It is well noted that Zn is integral part of antioxidative enzymes (superoxide catalase and dismutase), which regulates ROS generation in plant cells and is used by polymerase, kinase, dehydrogenase, and phosphatase enzymes in the photosynthesis process [72]. During the stress conditions, Zn can modulate the cellular redox and antioxidant defence system. In a recent study, it was shown that ZnO NPs with the combination of salicylic acid improved the activities of CAT, POX, and SOD in rice under As stress condition [73]. Based on the cellular importance of Zn, the Zn-based nano-fertilizer could enhanced the effectiveness of micronutrients applied in agriculture [74].

TiO_2_ NPs, however, require a higher dosage of application. The lower dose of TiO_2_ either causes a slight improvement in chlorophyll content or this happens in a dose-dependent manner. A hybrid photosynthetic apparatus prepared with chloroplasts coupled with a higher dose of TiO_2_ NPs has been studied to enhance the photosynthesis by improving the electron transfer rate in the photosystems; however, its practical application is still subject to approval for field application. Ag NPs exhibit effects similar to ZnO NPs that requires lower doses, and Au NPs have worked well with a higher dosage. However, for using Ag NPs and Au NPs, cost effectiveness could be a major issue.

The above studies are conclusive for the use of ZnO NPs that have shown excellent results at lower concentrations and are believed to have practical aspects for field application, in addition to their lower ecotoxicological implications, especially for green synthesized NPs inputs [75].

## 3. Nanobionics to Improve Nutrient Use Efficiency

Even though the soil is nutrient-rich, crops are often unable to effectively utilize those nutrients, because modern agriculture completely relies upon the fertilizers that can avail the macro and micronutrients such as N, K, Mg, P, S, Ca, etc., and Cu, Mn, Fe, B, Cl, Mo, and Zn, etc., respectively [76,77]. However, these commercial and chemical fertilizers have limits, and they can only provide 30 to 50% of total crop yield nutrition [78]. NUE is very much dependent on the soil characteristics (physicochemical properties), vaporization of chemical fertilizers and their leaching and also on the physicochemical characteristics of the fertilizers [79]. The cost of production in agriculture surges with the lowered NUE, and that ultimately affects the lower-income group farmers as well as stakeholders. Fertilizers with lower NUE also affect the environment and ecosystems, as their utilization is more compared to fertilizers with higher NUE [80]. The best agriculture practices must include fertilizers with greater NUE so that they can be cost effective and have minimal environmental impacts.

**Figure 1:**
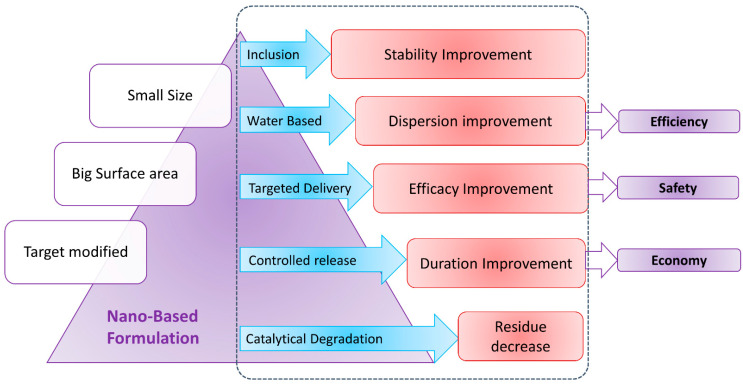
Merits of different types of formulation of nanobionics involved in enhancing the nutrient use efficiency of agrochemicals.

Nanobionics have emerged as a great means that can improve the NUE by employing a smart delivery system (Figure 1). Such nanocarriers are capable of performing controlled release of the ingredient triggered by various environmental conditions, and can load tiny amounts of fertilizers, thereby widening their distribution [81]. The release of nutrients depends on the types of carriers, choice of fertilizers and design specific to the stimuli.

**Figure 2:**
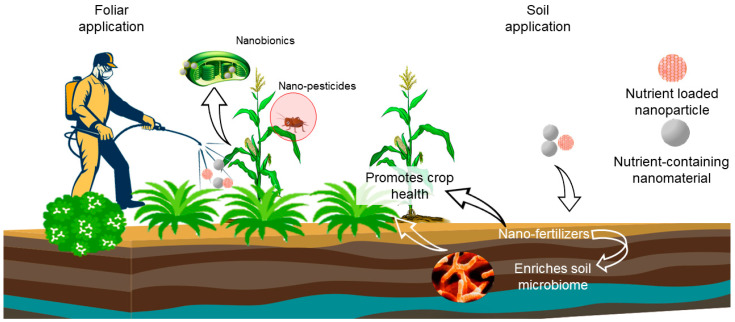
Illustration of the use of nanobionics for soil and foliar applications to improve the crop production.

The release even can be pre-planned as per the requirements and executed through some external stimuli. These stimuli can be pH, temperature, humidity, molecular recognition, light, and ultrasonic shock waves [82,83,84]. Such controlled release of ingredients acts as a function of time or interaction with an appropriate environment, as per the strategy of the nano-encapsulation (Figure 2).

Owing to the promising applications of stimuli-responsive NPs for agricultural application, in this context, few studies have reported the controlled release of active ingredients along with extended-release duration, which are vital to their field application. Polymeric ZnO NPs that were intended to work with agricultural soils at specific pH and organic matter exhibited a slow-release pattern of Zn in the soil with time. This demonstrated that the release of Zn was in accordance with the requirement of *Zea mays* L., thereby preventing excess Zn bio-availability in the soil and therefore ecotoxicity [85].

Engineered SiO_2_ NPs (SiO_2_: NPs as the core, chitosan: semi-permeable coating, and Sodium Alginate and Kaolin: outermost superabsorbent coating) ((K-SA-CS-SiO_2_ NPs) loaded with NPK have exhibited sustained release of nutrients for six months at room temperature. They have also helped in fighting drought and salinity stress [86]. SiO_2_ NPs with polyacrylate emulsion used for the formulation of controlled release urea formulation (CRUF) along with polyacrylate/silica hybrid were found to be significant. The 1.0 wt.% SiO_2_ used with polyacrylate emulsion reduced the solubility of CRU 38.3 wt.% to 2.2 wt.%. The release time was also extended from 8 to 27 days [87].

Apart from metal and slow-release type NPs, the pH-responsive NPs composed of poly[(meth)acrylic acid] and poly[N,N dimethylaminoethyl(meth)acrylate] are recognized to be functional in a range of physiological conditions. They have been reported to be promising for agricultural application due to their nondegradable all-carbon backbones [88]. Other NPs are also being studied for assessing the potential material for nano-carriers composed of chitosan, polyacrylic acids and zeolites [14,89,90].

A wide range of commercial preparations of nano-fertilizers are available around the globe, such as nitrogen (IFFCO Nano Urea, IFFCO, New Delhi, India), phosphorus (TAG Nano Phos, SK Organic Farms, Chennai, India), potassium (NanoMax Potash, JU Agri Sciences, New Delhi, India), potassium and phosphorus (Fosvit K30, Kimitec Group, Almería, Spain), zinc (Geolife Nano Zn, Geolife Agritech India Ltd., Mumbai, India, Silvertech Kimya Sanayi ve Ticaret Ltd., Istanbul, Turkey and Nano Zinc Chelate Fertilizer, AFME Trading Group, Canary Wharf, UK), and iron and magnesium (Nubiotek^®^Hyper Fe+Mg, Bioteksa, Chihuahua, Mexico) [91].

## 4. Foliar Application of Nanoparticles

### 4.1. Adhesion to the Foliar Surface

Foliar application of NPs has been documented to be very effective and efficient for crop production. It can reduce the leaching of agrochemicals, thereby reducing the impacts on the environment and ecosystem [58]. Controlled and targeted delivery ensures localized delivery of the active ingredients on the crops for effective utilization and improved efficiency [92]. Cuticle and epidermal tissues of the foliage have distinct features and environmental conditions [93]. In the kingdom Plantae, amphistomatic, epistomatic lamina and hypostomatic are differentiations that are controlled by environmental conditions such as temperature and humidity [94]. The design of NPs should be focused to target the types of foliage morphology and chemical characteristics. Usually, the leaf cuticles have an abundance of wax layers that enable low surface energy, which results in increased contact angle of the water, hence preventing wetting [95]. Therefore, to ensure the sufficient adhesion of the NPs after a foliar application, the design should be preferred that can consider their shape, size and surface chemistry [96]. The most preferred shape of the carriers are spherical; however, rod- and platelet-shaped carriers can be given preference for the foliar application pertaining to their better contact areas [96].

A report on a poly-lactic acid (PLA) carrier of 450 nm loaded with abamectin (an insecticide) was studied for improving the hydrophobicity and positive surface groups. NPs with functional groups such as acetyl, amine or carboxylic acid were greatly helpful in adhesion to the cucumber leaves, and the order of adhesion was observed to be amine > acetyl > carboxylic acid. Particles modified with the amine functional group showed the strongest adhesion, which retained itself even after multiple irrigations [97]. Nanoionics surfaces modified with tannic acid have also shown enhanced adhesion to foliage tissues for delivery of insecticide and fungicides [97,98]. The adhesion is greatly contributed by H-bonding brought about by OH groups present on the phenol rings, and 50% greater adhesion was achieved by the modified nanobionics compared to the unmodified surface. The modification with polyphenolic groups greatly increases the photostability of the active ingredient without disturbing the release event [99]. Other surface modifications such as carboxymethylcellulose with ethylenediamine [100], graphene oxide nanosheets with polydopamine [101], and styrene-methacrylic acid are also successful in improving the adhesion of NPs to the plants [102].

### 4.2. Uptake and Translocation within the Plant System

Plant uptake of NPs from the soil is achieved by roots that distribute and translocate up to the leaves. However, in the case of foliar application, the leaf to leaf translocation in distribution is common [103]. Translocation through the roots is a normal hydroponic phenomenon attributed to the plant vascular system; however, the uptake via leaves is important to foliar application and particulate delivery [104]. Uptake after the foliar application is greatly influenced by physicochemical properties of the NPs such as size, shape, and surface chemistry [105]. Studies have elaborated the understanding of plants’ mechanisms and distribution limits and their fate in the plant system after the uptake [106]. Internalization of NPs by leaves usually follows many routes. They enter through stomata via the symplastic or apoplastic transport system of the plants [107,108]. The apertures of stomata that are usually in micrometres allows spontaneous uptake of the NPs, and leaf mesophylls play a vital role in the transport and distribution. Smaller NPs can penetrate the junctions of leave cuticles [109]. As NPs are transferred from soil into plants, they are mobilized by apoplastic and symplastic mechanisms. It is reported that the apoplastic pathway supports radial distribution, which leads NPs to be directed toward the root core cylinder and vascular systems, as well as an upward tendency towards the aerial sections of the plant [110,111]. The apoplastic pathway is critical for the transport of NPs, whereas the Casparian strip prevents NPs from migrating radially in the endodermis of roots. The Casparian strip can be avoided by switching from the apoplastic to the symplastic pathway [112,113]. This pathway is more orderly and well-regulated than the other pathways that allow NPs to transit through the plant body. Once the NPs have reached the cytoplasm of the cell, the plasmodesmata aid in cell-to-cell migration [114].

A study using Au NPs of various sizes (3, 10 or 50 nm) and surface chemistry (treated with polyvinylpyrrolidone (PVP) and citrate) was performed to explore the role of physicochemical properties of the carrier NPs on interactions and translocation in the plant system. The study inferred that distribution in the plant was greatly affected by size and surface chemistry. The PVP-modified NPs showed better adhesion compared to citrate, which could not penetrate the cuticle for almost 15 days. However, the PVP-Au NPs were internalized and translocated up to the mesophyll cells. The smaller Au NPs (3, 10 nm) after foliar application were more persistent with respect to Au NPs of size 50 nm [96].

Another study that included the liposomes of 100 nm prepared from hydrogenated soybean L-α-phosphatidylcholine carrying Fe and Mg ions were greatly helpful in overcoming the acute nutrient deficiency of the tomato. The study reported up to one-third of the applied liposome penetrated the leaves and delivered the ions quickly compared to conventional treatment that penetrates 1% of the total free molecules applied in the same fashion. The liposomes were readily distributed to the plant system and detected in the roots after just 24 h of exposure [115].

The poly(ε-caprolactone) nanocapsules of 300 nm that carried atrazine were studied for leaf uptake after foliar application on mustard leaves. The nanocapsules remained active on the surface for 24 h after the application. After 48 h, they translocated to vessel elements and after 96 h of the treatment, they were found in the gaps between the mesophyll cells. The atrazine encapsulated when delivered in a site-specific manner demonstrated excellent herbicide activity even after being diluted 10 times [116]. Findings from the studies affirm that NPs that are used as a carrier must pass through several physiological barriers until they are taken up by the plants and translocated. Interfacial interactions between the foliar tissue and the nanobionics play a decisive role.

The impact of shape, geometry, and dimension of nanobionics are the factors that alter their uptake and translocation in the plant system, and they should be further systematically studied on different plants for their beneficial or adverse effects.

## 5. Major Concern and Future Prospects

The application of NPs in crop production is promising yet in the nascent phase. Both engineered and green synthesized NPs have been explored extensively for improving crop production and other agricultural advances. Considering the application of nanobionics in augmenting photosynthesis and enhancing NUE, it is certainly believed that NT has considerable potential to revamp conventional agriculture practices. However, several other applications of NPs have equal potential as nanobionics. For example, NPs can be used to provide abiotic and biotic stress tolerance, site-specific delivery of agrochemicals, and nano-sensors for precision agriculture. Nano-enabled products are now being developed and debated in the scientific community for their agricultural application. Nearly 9425 nano-based products have been developed so far, which was contributed by 64 countries and 2658 industries, among which 229 products are for agriculture developed by 73 industries and 16 countries (https://product.statnano.com/; accessed on 10 January 2021). In the coming time, the focus of NP research and application must be addressed on the development of more nano-enabled products that can address conventional agricultural issues in an intelligent way.

However, with mere development of products, other aspects pertaining to the application of NPs in agriculture must be deliberated in the future: (a) guidelines for manufacturing, handling, and application, (b) crop-specific dose of application, (c) availability in the environment and their ecotoxicological concerns, and (d) toxicological aspects in food chains, including humans.

With the growing application of NPs their release in soil and water is gradually becoming inevitable. Deliberate application of NPs on the crops may lead to accumulation in the soil, thereby uptake by vital soil microorganisms and lower organisms is useful to agriculture. This may affect total organic content, nutrient cycling, biogeochemistry of the crop field and the overall ecosystem [117,118]. Metal-based NPs are most likely to release their constituents in the soil as elemental or ionic forms that can be used as nutrients (in the case of NPs of Cu, Mg, Mn, Se, Zn, etc.); however, the NPs of Ag, As, Pt, Ti, etc., that are equally promising in crop production, may accumulate after continuous and repeated applications and affect the environment negatively [119,120,121]. This can affect the germination of seeds as well as root and shoot development, thereby affecting the biomass and yield of the crops. NPs such as Ag NPs have been analysed in the soil, surface water, and sediment [59,121,122,123]. The accumulated NPs are transported via interfaces of soil particles and affect the beneficial soil microbes undesirably [118,123].

On the one hand, NPs have potential applications in improving crop production, and on the other hand, their repeated application causes a growing threat to agricultural soil and crops to hamper crop growth [6]. Therefore, further studies that can define and ensure the crop/disease/soil specific dosage, mechanism of internalization, and translocation and distribution in the plant system are prerequisites and challenges for this decade [8].

The ecotoxicological and toxicological aspect is an important concern that should also be dealt with in utmost tenaciousness. Once NPs enter the environment they undergo several modifications, such as dissolution, binding with soil/sediment particles, sulfidation aggregation/agglomeration, etc., and these modifications are key for assessing the accumulation in soil and biota, precipitation and also toxicity [124]. Other studies have also reported several NPs for the trophic transfer from green algae to fish by a two-stage food chain [125], in river snails (*Cipangopaludina chinensis*), and Chinese muddy loaches (*Misgurnusmizolepis*) [126]. These studies could be alarming as well as trendsetters, followed by which further studies should delineate more sensitive biotas. Interaction of NPs with key protein and biomarkers (also from humans), genotoxicity, toxicodynamics and toxicokinetics are the key areas where more data generation is certainly required.

The feasibility of acceptance of NP-based formulations is one of the biggest challenges identified ahead in this decade. No matter how great the potential of NP-formulations, if they are not cost effective, it will be hard to find a market among the developing and underdeveloped countries where the livelihoods of the majority of the farmers are dependent on limited land resources and smallholders. Precision farming and the use of nanobionics may imply sensors and gadgets that would be far beyond the reach of smallholders.

To sum up, the sustainable development that the world is striving for should be inclusive. If such development can improve crop production, at the same time it is required to protect the natural resources (land, water and forests), biota, and also human beings. To seek and achieve the “Zero Hunger SDG 2030”, research planning should be ahead of the times to protect from the aftermath and adverse consequences, unlike the implementation of the use of chemical pesticides that have caused extensive damage to the environment and continue to persist in damage to environmental health. Application of NPs by the nanobionics approach would certainly shape and build the future of crop production, but understanding all the aspects of their practical application is necessary before they unleash their harmful impacts on the environment and human health.

## 6. Conclusions

Nanobionics can modulate photosynthesis by improving the light absorption up to the near-infrared region, improving the number of thylakoids and chlorophyll content that overall boosts the biomass and crop production. Nanocarriers that respond to external stimuli such as pH, temperature, humidity, pathogens, or even manmade stimuli to deliver agrochemicals in a controlled release are promising for improving the NUE and minimizing wastage. These two approaches are considerable for cost-effective crop production and sustainable agriculture. They can together improve conventional agriculture practices to a smarter and affordable agriculture. Thus, to achieve the “Zero Hunger” goal of UN SDG by 2030 there is an urgent need for a paradigm shift in conventional agriculture practices that can effectively be brought about using emerging and smart NPs such as nanobionics.

However, the increasing application of NPs in agriculture, where they directly interact with the environment, poses threats to aquatic and terrestrial species as well as humans. Deliberate application may lead to the accumulation of NPs or their constituent elements in the soil [118], and that could affect physicochemical characteristics as well as fertility of the soil. Such accumulation can also affect the plant health brought about by NP-induced oxidative stress, cell death and genotoxicity, thereby affecting the biomass of the plant, chlorophyll content, and resulting in diminished yield. Moreover, beneficial microbes and lower soil animals (e.g., nematodes and earthworms) who ensure better nutrient cycling and maintain soil health are also affected.

Colloidal microstructures or aggregates of the soil particles are responsible for carrying the NPs from the soil to water bodies, where they are taken up by lower trophic aquatic species and then biomagnified. NPs affect the biological system by interfering in the biochemical processes at the molecular as well as tissue organ levels. The ROS-induced oxidative stress, followed by DNA damage, apoptosis, and altered protein functions, are key events by which NPs exert toxicological effects.

## Figures and Tables

**Table 1:** Application of various nanoparticles as nanobionics and their impact on photosynthesis.

Type of NPs	Size (nm)	Shape	Plants	Effect on Photosynthesis	References
ZnO NPs (10 mg L^−1^)	20–30 nm	spherical	*Triticum aestivum* L.	Enhanced photosynthesis by enhancing the photosynthetic pigments chlorophyll a, chlorophyll b, and total chlorophyll content Increased the process of water splitting complex at donor side of PS-II (Fv/Fo)	[56]
25, 50, 75, 100 and 150 μM Ag NPs and AgNO_3_	50 nm	nanospheres	*Nicotiana tabacum* L.	AgNP and AgNO_3_ enhanced the chlorophyll content that significantly lowered values of relative electron transport rate Coefficient of photochemical quenching, implying an inhibition of the electron transport chain	[51]
100 mg kg^−1^ Ca(NO_3_)_2_ in the soil and 50, 75, and 100 mg kg^−1^ CaNPs in the soil	80 nm	particle meshwork	*Moringa oleifera* L.	Enhanced physiological parameters of the plant Photosynthetic ability was significantly enhanced at 50 mg CaNPs kg^−1^ soil	[57]
SiO_2_ and TiO_2_ NP foliar spray 0, 5, 10, 15, and 20 mg L^−1^			*Oryza sativa* L.	Allowed plants to nurture in contaminated soil with Cd metals It reduced the uptake of Cd and protects the antioxidant system	[58]
TiO_2_ (Aeroxide^®^ P25: A80:R20, 21 nm)-NPs 5, 50, and 150 mg L^−1^	60 nm	spherical	*Triticum aestivum* L.	Reduced the chlorophyll content and hampered the efficiency of PS-II Did not affect the RuBisCo or total sugar content (TSS)	[45]
Al_2_O_3_ NPs 5, 25, and 50 mg L^−1^ Suspension	~150 nm		*Triticum aestivum* L.	Caused oxidative stress and affected the pigments of the photosystem Reduced roots and shoots in all treatments	[46]
Diclofop-methyl (an herbicide) and citrate-coated Ag NPs Diclofop-methyl (0.5, 1.0 and 1.5 mg L^−1^) and Ag NPs (0.1, 0.5 and 1.0 mg L^−1^) on MS media	63–67 nm		*Arabidopsis thaliana* L.	Led to the oxidation of chlorophyll and the apparatuses of photosystem It also affected the growth of the plant Diclofop-methyl affected the Ag^+^ released from Ag NPs solutions	[59]
Au NPs	10–14 nm		*Robinia pseudoacacia* L.	Induced the reabsorption of photoemission from PS-II, improved photosynthesis	[41]
ZnO NPs 0, 50, 100, 200, 250, and 300 mgL^−1^	<50 nm		*Arabidopsis thaliana* L.	Zn NPs prevented the biogenesis of chlorophyll and affected PS-I thereby reducing the rate of photosynthesis	[60]
CuO NP 1000 mg L^−1^	<50 nm	spherical	*Oryza sativa* L.	Reduced the number of thylakoids in the granum Stopped the expression of key proteins of the PS-I Higher concentration destroyed PS-II	[61]
Fe_3_O_4_, Co_0.2_Zn_0.8_Fe_2_O_4_, and Co_0.5_Zn_0.5_Fe_2_O_4_, 0, 12.5, 25, 50, 100, 200 or 400 mg L^−1^	5–10 nm	spherical	*Lemnagibba gibba* L.	Induced the production of ROS that destroys chlorophyll and components of PS-II, thereby completely stopping photosynthesis	[62]

**Table 2:** Mostly studied nanoparticles and their impact on chlorophyll content, photosynthesis modulation and response to stress conditions.

Types of NPs	Effective Concentration	Size and Shape	Plants	Impact of Chlorophyll Content	References
Ag NPs	40 mg L^−1^		*Trigonella foenum-graecum* L.	Significantly enhanced chlorophyll a and b along with carotenoids, and other pigments	[47]
	50 mg L^−1^ (green Ag NP)	20 nm	*Cucumis sativus* L.	Significantly enhanced total chlorophyll a and b; however, carotenoid content was decreased	[48]
	10, 25, and 50 mg L^−1^ (green Ag NP)	<90 nm, spherical	*Eschscholzia californica* Cham	Chlorophyll a content by 39.7%, 35.1% and 38.7%, respectively, 25 mg L^−1^ bAg NPs led to a higher increase by 45.6% in total chlorophyll content over the control Decreased by 48.3% followed at 100 mg L^−1^	[49]
	20 mM	50–100 nm	*Pennisetum glaucum* L.	(enhanced chlorophyll a by 10% and chlorophyll b by 24%)	[63]
	60 mg L^−1^ (green Ag NPs)	100 nm, spherical	*Helianthus annuus* L.	Significantly increased chlorophyll content	[50]
Au NPs	200 μM Au NPs (Melatonin in the form of Au NPs)	40 nm, spherical	*Oryza sativa* L.	Treated on the hydroponic system Restored chlorophyll biosynthesis (85.1%) that was affected by Cd (51.6% decrease) Cd uptake significantly decreased by 33.0% and 46.2%	[64]
	0.1–1.0 mg mL^−1^ (aspartate-capped AuNPs, BSA-capped AuNPs and sodium citrate-capped AuNPs)	20–45 nm, Spherical	*Vigna radiata* L.	Improved photon absorption in the light-harvesting molecular complexes The highest concentration of sodium citrate-capped AuNPs significantly improved the total chlorophyll content	[65]
	300 mg L^−1^ (green Au NPs) On salt stress condition	27 nm, spherical	*Triticum aestivum* L.	Chlorophyll contents were decreased by 49.11% in salt stress condition 77.05%, 60.16% and 72.34%, respectively, when supplemented with AuNP with NaCl	[66]
	100 mg L^−1^ Astaxanthin in the form of Au NPs (Ast-Au NPs) (Cd stressed)	52.5 ± 4.3 nm, spherical	*Triticum aestivum* L.	The presence of Cd in the culture medium diminished the total chlorophyll content; however, treatment with 100 μg/mL Ast-Au NPs restored the normalcy	[67]
TiO_2_ NPs	25, 50 and 100 mg L^−1^	50–100 nm, spherical	*Solanum lycopersicum* L.	A slight increase in total chlorophyll content with 25, 50 and 100 µg/mL; however, reduced with the application of concentration 200 and 400 µg/mL	[52]
	100–500 mg kg^−1^ (Cd-contaminated soil)	15–40 nm	*Trifolium repens* L.	Slightly increased Chl a and Chl b contents Compared to control 43.27% increase in total chlorophyll content was achieved at 250 mg kg^−1^ TiO_2_ NPs along with PGPR application	[54]
	500 mg L^−1^ TiO_2_ in conjunction with 210.87 mg L^−1^ chloroplast (a hybrid semiartificial photosynthesis system)	2.0−5.0 nm, crystalline	*Lactuca sativa* L.	The highest electron-transfer rate from PS-II to PS-I during the photosynthetic process	[55]
	20 and 40 mg L^−1^ per kg of the soil (green TiO_2_ NPs)	spherical	*Triticum aestivum* L.	A dose-dependent increase in chlorophyll content in chlorophyll content	[53]
ZnO NPs	10 mg L^−1^ of ZnO NPs by seed priming for 18 h	20–30 nm, spherical	*Zea mays* L.	Compared to control Chl a had a 48% increase and Chl b had a 50% increase reported Total Chl content was increased by 49% as compared with the control	[56]
	50 mg L^−1^ ZnO NPs, foliar application (salt-stressed)		*Solanum lycopersicum* L.	Total chlorophyll concentration was enhanced by 34% with respect to control The rate of photosynthesis, stomatal conductance, and internal CO_2_ concentration was also enhanced by 34%, 32%, and 35%, respectively	[68]
	20 mg L^−1^ ZnO NP by seed priming (arsenic-stressed)	20–30 nm, spherical	*Oryza sativa* L.	Total chlorophyll concentration was increased by 40.1% The higher dose (100 and 200 mg L^−1^) did not affect the chlorophyll concentration significantly	[69]
	500 mg L^−1^ ZnO NPs by seed priming for 24 h (cobalt-stressed)	20 nm, crystalline	*Zea mays* L.	ZnO NPs chlorophyll content, photosynthetic efficiency and biomass accumulation Plants were also protected from cellular ultra-cellular structure damage under Co stress	[70]

## Data Availability

Not applicable.
